# Pax4 is not essential for beta-cell differentiation in zebrafish embryos but modulates alpha-cell generation by repressing arx gene expression

**DOI:** 10.1186/1471-213X-12-37

**Published:** 2012-12-17

**Authors:** Joachim Djiotsa, Vincianne Verbruggen, Jean Giacomotto, Minaka Ishibashi, Elisabeth Manning, Silke Rinkwitz, Isabelle Manfroid, Marianne L Voz, Bernard Peers

**Affiliations:** 1 From Unit of Molecular Biology and Genetic Engineering, Giga-Research, University of Liège, 1 avenue de l'Hôpital B34, Sart-Tilman B-4000, Belgium; 2 From Developmental Neurobiology and Genomics, Brain and Mind Research Institute, University of Sydney, Camperdown, NSW, 2050, Australia

**Keywords:** Insulin, Glucagon, Pancreas, pax4, Arx, mRNA export, Development, Zebrafish

## Abstract

**Background:**

Genetic studies in mouse have demonstrated the crucial function of PAX4 in pancreatic cell differentiation. This transcription factor specifies β- and δ-cell fate at the expense of α-cell identity by repressing *Arx* gene expression and ectopic expression of PAX4 in α-cells is sufficient to convert them into β-cells. Surprisingly, no *Pax4* orthologous gene can be found in chicken and Xenopus *tropicalis* raising the question of the function of *pax4* gene in lower vertebrates such as in fish. In the present study, we have analyzed the expression and the function of the orthologous *pax4* gene in zebrafish.

**Results:**

*pax4* gene is transiently expressed in the pancreas of zebrafish embryos and is mostly restricted to endocrine precursors as well as to some differentiating δ- and ε-cells but was not detected in differentiating β-cells. *pax4* knock-down in zebrafish embryos caused a significant increase in α-cells number while having no apparent effect on β- and δ-cell differentiation. This rise of α-cells is due to an up-regulation of the Arx transcription factor. Conversely, knock-down of *arx* caused to a complete loss of α-cells and a concomitant increase of *pax4* expression but had no effect on the number of β- and δ-cells. In addition to the mutual repression between Arx and Pax4, these two transcription factors negatively regulate the transcription of their own gene. Interestingly, disruption of *pax4* RNA splicing or of *arx* RNA splicing by morpholinos targeting exon-intron junction sites caused a blockage of the altered transcripts in cell nuclei allowing an easy characterization of the *arx*- and *pax4*-deficient cells. Such analyses demonstrated that *arx* knock-down in zebrafish does not lead to a switch of cell fate, as reported in mouse, but rather blocks the cells in their differentiation process towards α-cells.

**Conclusions:**

In zebrafish, *pax4* is not required for the generation of the first β- and δ-cells deriving from the dorsal pancreatic bud, unlike its crucial role in the differentiation of these cell types in mouse. On the other hand, the mutual repression between Arx and Pax4 is observed in both mouse and zebrafish. These data suggests that the main original function of Pax4 during vertebrate evolution was to modulate the number of pancreatic α-cells and its role in β-cells differentiation appeared later in vertebrate evolution.

## Background

In vertebrates, the pancreas plays a fundamental role in nutritional homeostasis through the secretion of various enzymes and hormones by acinar and endocrine cells, respectively. The endocrine cells form aggregates, called islets of Langerhans, and are composed of five cell subtypes (α-, β-, δ-, ε- and PP-cells) each producing a distinct hormone (glucagon, insulin, somatostatin, ghrelin and pancreatic polypeptide, respectively). All endocrine pancreatic cells differentiate during embryogenesis from progenitor cells specified in the dorsal and the ventral pancreatic buds, two protrusions that emerge from the endodermal embryonic gut [1]. This differentiation process is controlled by a panoply of transcription factors [2,3], such as PDX1 and PTF1a acting at early stages to specify the pancreatic progenitors [4-6], then the HLH factors NGN3 and NEUROD inducing the endocrine precursors [7,8], and the homeodomain factors NKX2.2, NKX6.1, PAX6, PAX4 and ARX controlling the formation of the five endocrine cell subtypes [9-14].

In mouse embryos, *Pax4* gene expression first appears in endocrine precursors and then is detected transiently in numerous differentiating β-cells and occasional α-cells [15,16]. *Pax4* expression seems to switch off upon terminal β-cell maturation [17,18], although some studies have reported expression in adult β-cells [19,20]. PAX4 has at least two functions in the differentiation of murine pancreatic cells. First, it favours the fate of the endocrine precursors toward the β- and δ-cell fate while repressing the α-cell lineage. Indeed, *Pax4* mutant mice display a lack of δ-cells, an almost complete loss of β-cells and an increase in α-cells [10]. This first role is due, at least in part, to the repression by PAX4 of the *Arx* gene, which encodes for an aristaless homeodomain factor and is absolutely required for the differentiation of α-cells [12]. Inversely, ARX is also able to repress *Pax4* gene expression and the *Arx* mutant mice have no α-cells and an increase of β-and δ-cells. So, the balance of α-cells versus β-/δ-cells in pancreatic islets is controlled in mouse by an antagonistic action of the two homeodomain factors ARX and PAX4. While PAX4 favours the δ- and β-cell fate, it has no role per se in δ-cell differentiation; indeed, the double *Arx*-/-; *Pax4*-/- mice have no α- and almost no β-cells but display a strong increase in δ-cells [21]. Furthermore, ectopic expression of PAX4 in endocrine precursors or even in α-cells of transgenic mice is sufficient to force their conversion into β-cells, but not in δ-cells [19]. These data demonstrate the essential role of PAX4 in β-cell differentiation. Surprisingly, while birds and amphibians possess pancreatic β- and δ-cells, no *Pax4* gene has been reported in these organisms and examination of the chicken and Xenopus *tropicalis* genomic sequences indicates a lack of *Pax4* ortholog in these two vertebrates. A recent phylogenetic study strongly suggests that the *Pax4* gene is derived from a duplication Pax6/eyeless gene which probably occurred at the so-called two-round (2R) genome duplication in early vertebrates [22]. This ancient *Pax4* gene could have been lost in birds and some amphibians. In contrast, fish have the *pax4* orthologous gene but its function in pancreatic cell differentiation is still unknown.

The lack of *Pax4* gene in chick and Xenopus tropicalis is quite puzzling and raises the question about the pancreatic function of PAX4 protein during early vertebrate evolution and notably in fish. Two hypotheses can be proposed: i) PAX4 was important for β- and/or δ-cell differentiation in the first vertebrate organisms but the loss of *Pax4* gene in birds and amphibians has been compensated by another transcription factor or by others mechanisms, ii) the role of PAX4 in β- and δ-cell differentiation appeared later in vertebrate evolution. To tackle this question, we examined in the present study the expression and function of *pax4* in zebrafish and investigated the regulatory links with the *arx* zebrafish orthologous gene. We show that *pax4* is dispensable in zebrafish for the differentiation of the β-cells deriving from the dorsal bud, but has a role in the reduction of α-cells. This role is mediated by repression of *arx* gene whose essential function in α-cell formation appears to be maintained between zebrafish and mammals. In addition, our data uncover a so far unappreciated autorepression of *pax4* and *arx* genes. We also analyzed the fate of endocrine cells after *pax4* or *arx* knock-down. In light of our data, we propose a model where the main role of Pax4 during the initial phases of vertebrate evolution was to modulate the number of endocrine α-cells.

## Results

1) Expression of zebrafish *arx* and *pax4* genes during pancreatic development.

The zebrafish *pax4* gene sequence was identified on chromosome 4 by using the TBLAST program and the corresponding *pax4* cDNA was amplified by RT-PCR and 3’-RACE. Sequence comparison of the deduced zebrafish Pax4 protein with the human and mouse PAX4 proteins shows that the homeodomain and the paired domain are well conserved while the C-terminal domain is highly divergent (see Additional 1: Figure S1). As the neighbouring genes located to the 5’ side from *pax4* gene are the same in zebrafish, in mouse and in human (*snd1 gene: staphylococcal nuclease domain containing 1*; and *lrrc4 gene: leucine rich repeat containing 4*), this indicates that the zebrafish *pax4* gene is the actual ortholog of the mammalian *PAX4* genes (see Additional file 2: Figure S2). The zebrafish proteins showing the closest similarity with the deduced Pax4 zebrafish protein are Pax6a and Pax6b. Sequence comparison of vertebrates PAX4 and PAX6 proteins indicate that 13 amino acid positions are identical among the vertebrate PAX4 proteins while distinct from the PAX6 proteins (see Additional file 1: Figure S1). All together, these data indicate that, while the sequence of *PAX4* genes were much less conserved during vertebrates evolution compared to the *PAX6* genes, these *PAX4* genes are actual *PAX4* orthologs.

**Figure 1:**
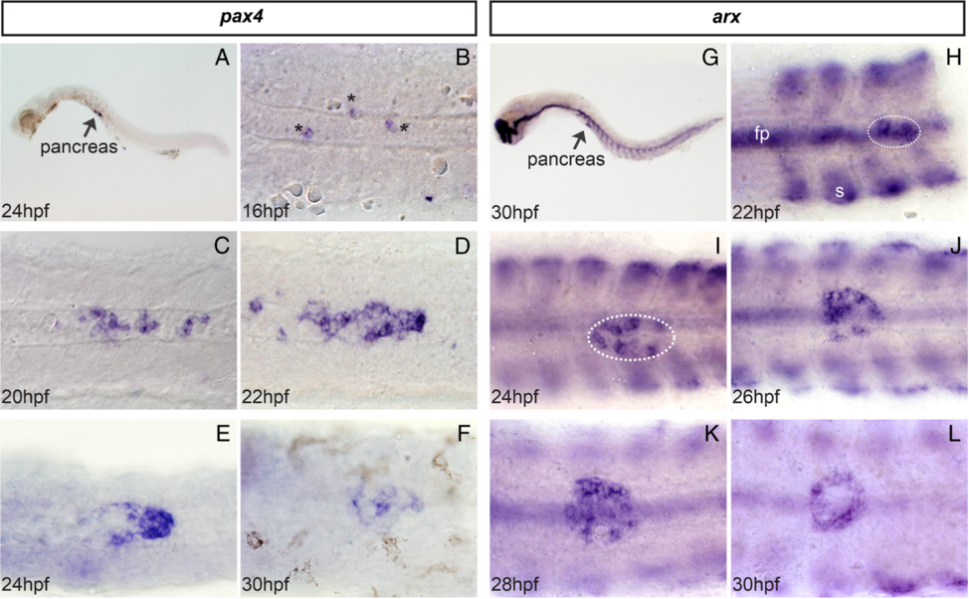
***pax4 *and *arx *are expressed in the pancreatic dorsal bud in zebrafish embryos. **Whole-mount *in situ *hybridization (WISH) performed with *pax4 *(**A – F**) and *arx *(**G – L**) probes at different developmental stages (hpf : hours post-fertilization). (**A **and **G**) Lateral view, anterior to the left, original magnification 40x. (**B – F **and **H - L**) Ventral views, anterior to the left of yolk-free embryos, original magnification 400x. Dotted lines : dorsal pancreatic bud; fp: floor plate; s: somites.

The expression of zebrafish *pax4* was analyzed by whole-mount *in situ* hybridization (WISH) at various stages of development. As described for the murine *Pax4* gene, zebrafish *pax4* transcripts were detected only in the pancreatic region, the first *pax4+* cells appearing at the level of the dorsal pancreatic bud at around 16 hours post fertilization (hpf) (Figure 1A and B). *pax4* pancreatic expression reaches its maximal level around 22-24 hpf, then progressively decreases and becomes hardly detectable after 40 hpf (Figure 1C-F). Some *pax4*+ cells can occasionally be detected at larval stages (after 72 hpf) near the intra-pancreatic ducts (data not shown).

While Miura and collaborators have previously detected *arx* transcripts in the telencephalon, diencephalon, floor plate and somites of zebrafish embryos, they did not report *arx* expression in the pancreas [23]. However, our WISH using the *arx* probe revealed expression in the pancreatic dorsal bud from 22 hpf onward (Figure 1G-L). At 30 hpf, pancreatic *arx* staining displays the shape of a ring which is reminiscent of the disposition of glucagon-expressing α-cells (Figure 1L). The levels of *arx* gene expression decreases at later developmental stages but its expression can still be detected in pancreas of larvae and adult zebrafish (data not shown). Another gene related to *arx* was found in the zebrafish genome; this novel arx-like gene (named ENSDARG00000075896.2 in Ensembl website) is expressed in the CNS but not in the pancreas (data not shown).

**Figure 2:**
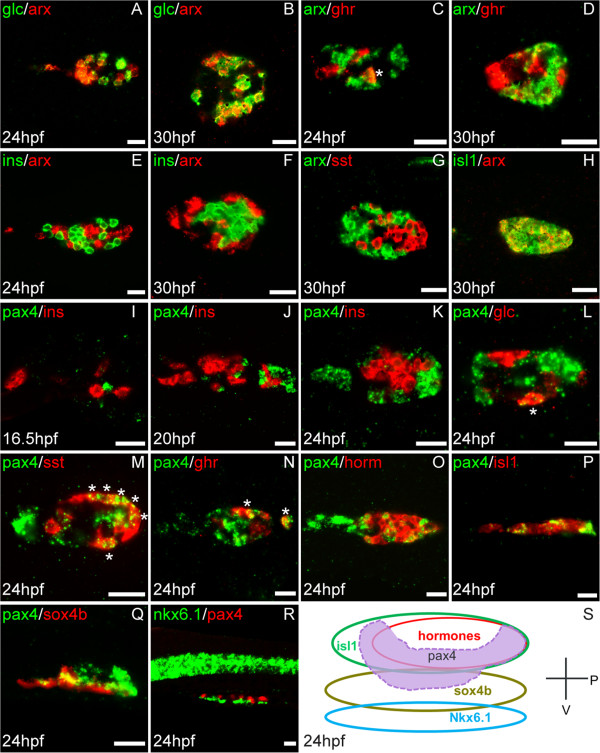
**Identification of *pax4 *and *arx *expressing cells by double fluorescent *in situ *hybridization. **All images are confocal optical sections of the dorsal pancreatic bud with anterior part to the left. The probes and the developmental stages are respectively indicated at the top and the bottom in each image. All views are ventral, except images **P**, **Q**, **R **and **S **which are lateral views. *arx *transcripts are detected in almost all *glucagon+ *(**A**, **B**), in some *ghrelin+ *(**C**, **D**) cells and in many *isl1+* cells (**H**); absence of co-staining between *arx *and *insulin* (**E**, **F**) and *somatostatin *(**G**). *pax4 *transcripts are not detected in *insulin*+ (**I **- **K**) and most glucagon+ cells (**L**). *pax4 *is expressed in many *somatostatin *(**M**) and a few *ghrelin *(**N**) expressing cells. Partial co-staining of *pax4 *probe with a cocktail of *insulin*, *glucagon*, *somatostatin* and *ghrelin *probes (horm)(**O**), with *isl1 *(**P**) and *sox4b *(**Q**) probes. Absence of co-staining between *pax4 *and *nkx6.1 *(**R**). **S**: Schematic representation of the multi-layer organization of the pancreatic dorsal bud at 24 hpf including *pax4 *expression. Scale bar = 20 μm. astérisks (*) indicate double positive cells.

In order to identify the pancreatic cell types expressing *arx* and *pax4*, fluorescent *in situ* hybridizations were performed with various pancreatic markers between 16 hpf and 30 hpf (Figure 2). *arx* transcripts were detected in all *glucagon*+ cells and in some *ghrelin*+ cells, but not in *insulin*+ cells and not in *somatostatin*+ cells (Figure 2A-H). At 30 hpf, all *arx*+ cells correspond to a subset of pancreatic post-mitotic *isl1*+ endocrine cells (Figure 2H). Thus, the expression profile of *arx* is quite similar in zebrafish and mouse, being mainly restricted to α-cells. In contrast, the pancreatic expression of *pax4* seems different in mouse and zebrafish. Indeed, while *Pax4* was detected in numerous differentiating β-cells in mouse embryos, we never observed co-localization of *pax4* and *insulin* transcripts in zebrafish embryos and larvae (Figure 2I-K). Most α-cells do not express *pax4* (Figure 2L), although some cells coexpressing *glucagon* and *pax4* could occasionally be found. In contrast, *pax4* was detected in many *somatostatin+* cells (in about 40% of δ-cells) and *ghrelin+* cells (about 15% of ε-cells) (Figure 2M and N). In order to determine whether *pax4* expression is restricted to hormone expressing cells, we performed a double staining of *pax4* with a cocktail of the four hormone probes (Figure 1O). At 24 hpf, about half of the *pax4*+ cells were not labelled by the hormones. Isl1 being expressed in all post-mitotic endocrine cells, we also analyzed the expression of *pax4* with this transcription factor. While many *pax4*+ cells were *isl1*+, some cells were found to be labelled only by the *pax4* probe (Figure 2P *).* To determine whether *pax4* is expressed in endocrine precursors, we next compared its expression with *sox4b*, a marker of these precursors in zebrafish [24,25]. Many *sox4*+ cells were found to express *pax4* (Figure 2Q). Finally, *pax4* was not detected in pancreatic nkx6.1+ progenitors which are located ventrally to the developing islet (Figure 2R) [24]. Taken together, these data indicate that *pax4* expression is restricted to pancreatic endocrine precursors and transiently in some differentiating endocrine cells such as δ- and ε-cells (see schematic diagram Figure 2S).

**Figure 3:**
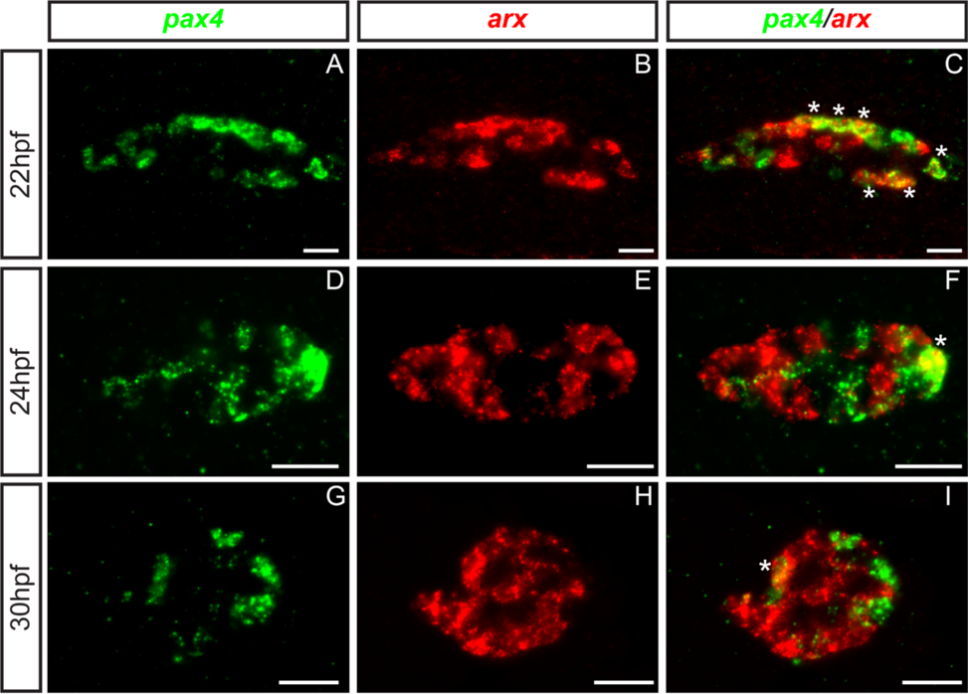
**Coexpression of *pax4 *and *arx *in some cells during pancreatic cell differentiation. **Ventral view of the pancreas area analyzed by double fluorescent WISH showing expression of *pax4* and *arx *at 22 hpf (**A **-** C**), 24 hpf (**D** -** F**) and 30 hpf (**G**-** I**). Confocal projection images with anterior part to the left. Scale bar = 20 μm. astérisks (*) indicate double positive cells.

We next investigated whether *pax4* and *arx* can be expressed in the same pancreatic cells. At 22 hpf, a substantial number of cells were found to contains both transcripts (Figure 3A-C); however, *arx* and *pax4* expression progressively segregates into distinct cells afterwards and only 1 or 2 cells per islet positive for the two transcripts were observed at 30 hpf (Figure 3D-I).

2) Function of *arx* and *pax4* in zebrafish pancreatic cell differentiation.

**Figure 4:**
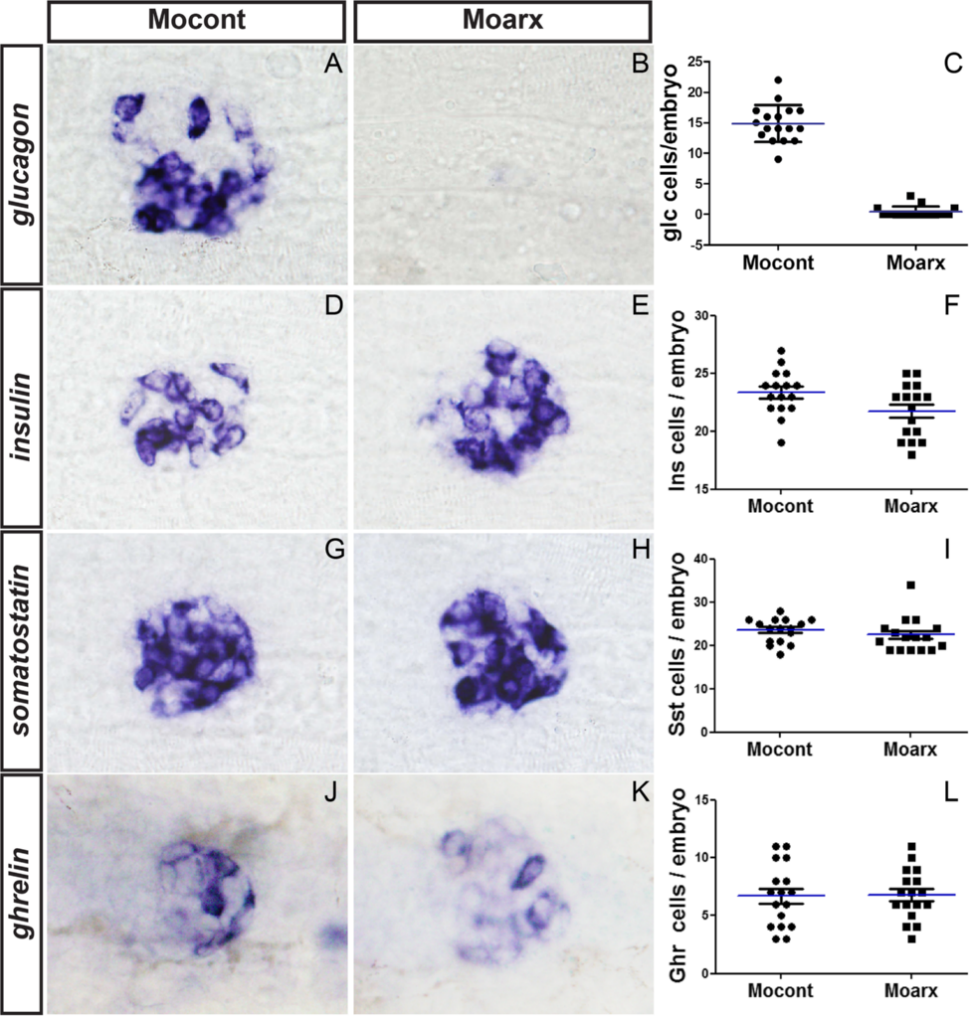
***arx *is required for the differentiation of α-cells. **(**A **- **H**) Ventral views of the pancreas area from embryos analyzed by WISH, anterior to the left. Pancreatic expression of *insulin *(**A**, **B**), *somatostatin *(**C**, **D**), *ghrelin *(**E**, **F**) and *glucagon *(**G**, **H**) in control morphants (**A**, **C**, **E**, **F**) and *arx* morphants (**B**, **D**, **F**, **G**) at 30n hpf. Quantifications (**I **- **K**) represent the number of positive cells per embryo for control morphants and arx morphants. Original magnification 400x.

**Figure 5:**
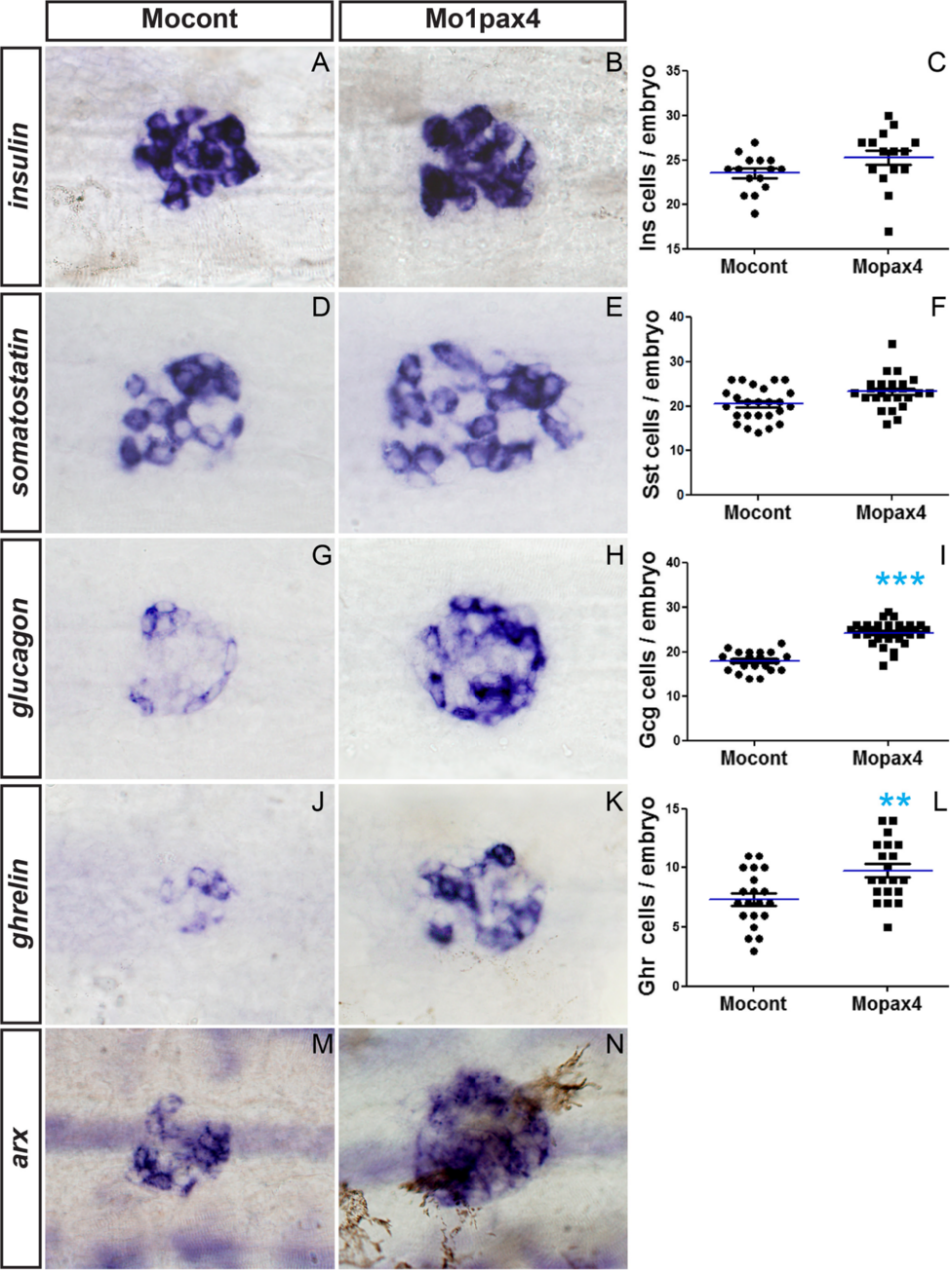
**Pax4 is not required for β-cell differentiation. **(**A **- **J**) Ventral views of the pancreas area from embryos analyzed by WISH, anterior to the left. Pancreatic expression of *insulin *(**A**, **B**), *somatostatin *(**C**, **D**), *ghrelin *(**E**, **F**), *glucagon *(**G**, **H**) and *arx *(**I**, **J**) in control morphants (**A**, **C**, **E**, **F**, **I**) and *pax4 *morphants (**B**, **D**, **F**, **G**, **J**) at 30 hpf. Quantifications (**K**, **L**, **M**, **N**) represent the number of positive cells per embryo for control morphants and *pax4* morphants. Asterisks (*) indicate that the difference between cell number in control morphants and pax4 morphants is statistically significant by Student’s *t*-test (**: *P*<0.003; ***: *P*<0.0001). Original magnification 400x.

To determine the function of the zebrafish *pax4* and *arx* genes in pancreatic cell differentiation, we injected antisense morpholinos designed to target exon-intron junctions in both pre-mRNA and to alter the splicing of these transcripts. To determine the efficiency of these knock-down, mRNA was extracted from these injected zebrafish embryos (named morphants) and from control embryos, and we analyzed the *pax4* and *arx* transcripts by RT-PCR (see Additional file 3: Figure S3). The morpholino Mo1pax4 targeting the exon2-intron2 boundary was highly efficient as injection of 6 ng was sufficient to block the removal of intron2 in almost all *pax4* transcripts, while Mo2pax4 targeting exon1-intron1 junction was less efficient and partially inactivate the splicing at that site. Thus, Mo1pax4 was used for the subsequent functional studies. Similarly, 2ng of Moarx morpholino was sufficient to block the splicing of intron2 for almost all *arx* transcripts (see Additional 3: Figure S3). Analysis of *arx* morphants revealed a complete loss of α-cells while the number of the other endocrine cell types was not significantly modified (Figure 4A-L). Injection of an unrelated morpholino (Mocont) or of the *arx* morpholino containing 5 mismatches in the exon2-intron2 junction site did not cause any changes in the number of pancreatic cells. In contrast to *arx* knock-down, injection of Mo1pax4 did not decrease the number of any pancreatic endocrine cell types (Figure 5); instead, a small but highly reproducible increase in *glucagon*+ cells number was observed (Figure 5G-I; p<0.0001) as well as a very slight increase in *ghrelin*+ cells (Figure 5J-L; p<0.003). Consistent with the increased number of α-cells, an increase in *arx*+ cells was also detected (Figure 5M-N) (28 ± 4 and 41 ± 5, respectively; n=30). Altogether, these data indicate that *arx* is required for α-cell formation, as described in mouse; however, *pax4* is not required for the generation of the first β-cells of zebrafish embryos but appears to modulate α-cell number.

3) Arx and Pax4 negatively autoregulate their own gene transcription.

**Figure 6:**
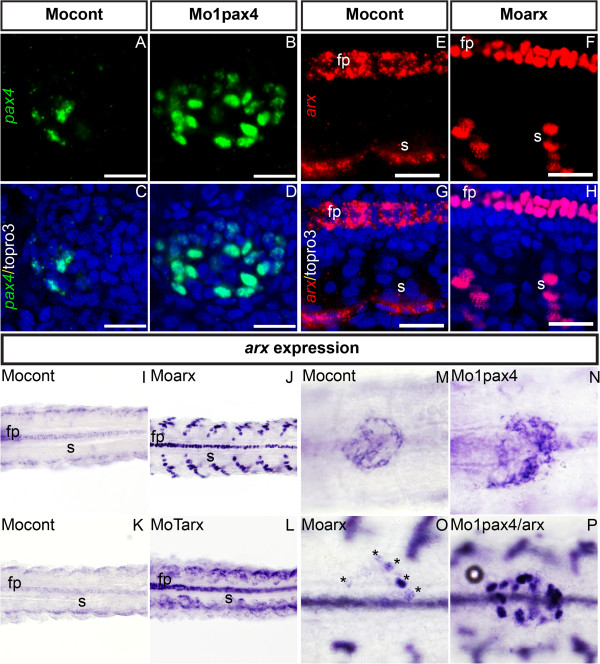
**Up-regulation and nuclear localization of *pax4 *and *arx *altered transcript. **(**A**–**D**) Confocal images of the pancreas area of 30 hpf embryos co-stained for *pax4 *mRNA (green) and Topro3 (blue) to visualize all cell nuclei, in control morphants (**A**-**C**) and *pax4* morphants (**B**-**D**). Scale bar = 20 μm. (**E**-**H**) Confocal images of the pancreas area of 30 hpf embryos analyzed by fluorescent WISH with *arx* probe (red) and stained for Topro3 (blue). *arx *expression in somites and the floor plate of control morphants (**E**, **G**) and of *arx *morphants (**F**, **H**). Scale bar = 20 μm. (**I**-**L**) Ventral view of WISH 30 hpf embryos showing upregulation of *arx *transcripts in somites and floor plate in Mo*arx *(**J**) and MoTarx (**L**) morphants. Original magnification 200x. (**M – P**) Ventral views of the pancreas area from embryos analyzed by WISH showing expression of *pax4 *in control morphants (**M**), *pax4* morphants (**N**), arx morphants (**O**) and double *pax4/arx *morphants (**P**). Original magnification 400x. Anterior part of all the embryos is in the left. Asterisks indicate *arx* positive cells. fp: floor plate, s: somite, cont: control morphant.

To determine whether transcription of the *arx* and *pax4* genes was affected after the inactivation of these two transcription factors, we next analyzed *arx* and *pax4* transcript levels by WISH in *arx* and *pax4* morphants, respectively (Figure 6). Surprisingly, while *pax4* mRNA was mainly cytoplasmic in control embryos as expected, the altered *pax4* transcripts were located in cell nuclei in Mo1pax4 morphants as revealed by colocalization with the nuclear marker TOPRO3 (Figure 6A-D). The intensity of fluorescence in nuclei was also higher in *pax4* morphants and the number of *pax4*-labelled cells was significantly increased. Similar data were obtained using the second Mo2pax4 morpholino, albeit with slightly less pronounced effects (data not shown). These data suggested that splicing alteration disrupted the transport of *pax4* transcripts from the nucleus to the cytoplasm and that a negative autoregulatory loop exists in the regulation of zebrafish *pax4* gene.

Interestingly, a similar phenomenon was observed for the *arx* gene. Indeed, while the level of *arx* staining was low and cytoplasmic in control embryos, it became nuclear and very strong in *arx* morphants (compare Figure 6E and F for fluorescent WISH; compare Figure 6I and J for visible WISH). This up-regulation was observed in all tissues expressing *arx* gene such as the floor plate, brain, spinal cord and somites, but not in the pancreas. Indeed, while about 20 pancreatic cells are labelled by *arx* probe in control embryos, very low staining was observed in a maximum of 5 pancreatic cells in *arx* morphants. To determine if the retention of arx transcripts in nuclei and the stronger staining in most tissues were caused by splicing alteration induced by the morpholino, we injected another morpholino (MoTarx) targeting the translation start site of *arx* mRNA. This morpholino did not cause a nuclear retention of *arx* transcripts, but it produced a strong increase in the level of arx transcripts in all tissues (compare Figure 6K and L), with the exception of pancreas where arx+ cells could hardly be detected (Compare Figure 6M and O, and see Additional 4: Figure S4 C,D). All together, these data indicate that i) splicing disruption is the cause of nuclear localization of transcripts and ii) a negative feed-back loop exists for Arx in all tissues, but is not detected in pancreas.

To verify that the up-regulation of *arx* transcripts in *arx* morphants is actually due to an auto-regulatory effect occurring at the level of *arx* gene transcription, we next used a zebrafish transgenic line in which GFP expression was driven by an enhancer of the human *ARX* gene. Performing a systematic enhancer-screening assay [26], a sequence was identified that regulated GFP in the central nervous system identical to the endogenous zebrafish *arx* gene (Ishibashi et al. manuscript in preparation). When the *arx* morpholinos were injected in one of the transgenic lines, a significant up-regulation of GFP was observed compared to control embryos (Additional 5: Figure S5). This confirms the existence of an inhibitory action of Arx protein on the expression of its own gene, this auto-regulation being either direct or indirect.

4) Antagonistic actions of zebrafish Arx and Pax4 in pancreatic cell differentiation.

**Figure 7:**
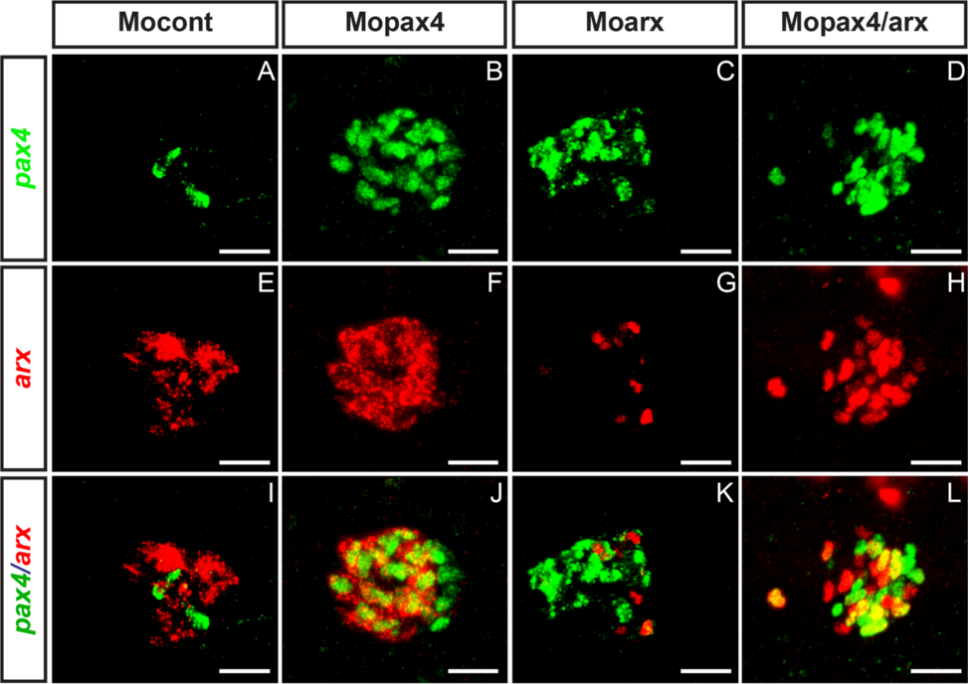
**Expression of *pax4 *and *arx *transcripts in single and double morphants. **Confocal projection of the *pax4 *domain (**A **– **D**), arx domain (**E **– **H**) and merged channels color (**I **– **L**) at 30 hpf in control morphants (**A**, **E**, **I**), *pax4 *morphants (**B**, **F**, **J**), *arx* morphants (**C**, **G**, **K**) and double *pax4/arx* morphants (**D**, **H**, **L**) analyzed by fluorescent WISH. Scale bar = 20 μm. Ventral view, anterior to the left.

The increase of *arx* transcripts in the somites and the central nervous system of *arx* morphants contrasts with the clear down-regulation of *arx* transcripts in pancreatic cells. This suggests the involvement of a pancreas-specific factor thwarting or impeding the negative feed-back loop. As Pax4 is described as a repressor of the *Arx* gene in mouse and is selectively expressed in pancreas, this prompted us to verify whether the down-regulation of *arx* transcripts in pancreatic cells of *arx* morphants was due to Pax4 action. To that end, we compared the expression of *arx* transcripts in the *arx* morphants and in the double *arx;pax4* morphants. We found that indeed, that the levels of the pancreatic *arx* transcripts were much higher in the double morphants (Figure 6, compare M, O and P). These data demonstrate that zebrafish Pax4 represses *arx* gene expression in zebrafish and is the factor thwarting the *arx* negative feed-back loop in the pancreas. To further analyse the antagonistic actions of Arx and Pax4, we determined the pancreatic expression of these two genes by fluorescent WISH in the single and double *arx;pax4* morphants at 30 hpf (Figure 7). In control embryos, *arx* and *pax4* transcripts were mostly detected in distinct cells at this stage. Following *pax4* knock-down, we detected an up-regulation of both *arx* and *pax4* transcripts and the nuclei of many *arx*+ cells were also co-labeled by the altered *pax4* transcripts (Figure 7B,F and J). In *arx* morphants, besides downregulation of *arx* gene transcription, increased number of *pax4*+ cells was noticed, with some of them expressing low amount of altered *arx* transcripts (Figure 7C,G,K). This co-expression was more obvious in the embryos injected with both *arx* and *pax4* morpholinos, as the two altered transcripts were strongly detected in many nuclei and around 50% of *arx*+ or *pax4*+ cells were co-labeled by the two probes (Figure 7D,H,L). In conclusion, as *arx* and *pax4* are expressed in distinct cells in control embryos while the two genes are co-transcribed in many cells in the double morphants, this confirms the mutual repression of these two factors on the each gene.

5) Characterization of Arx- and Pax4-deficient cells in zebrafish morphants.

**Figure 8:**
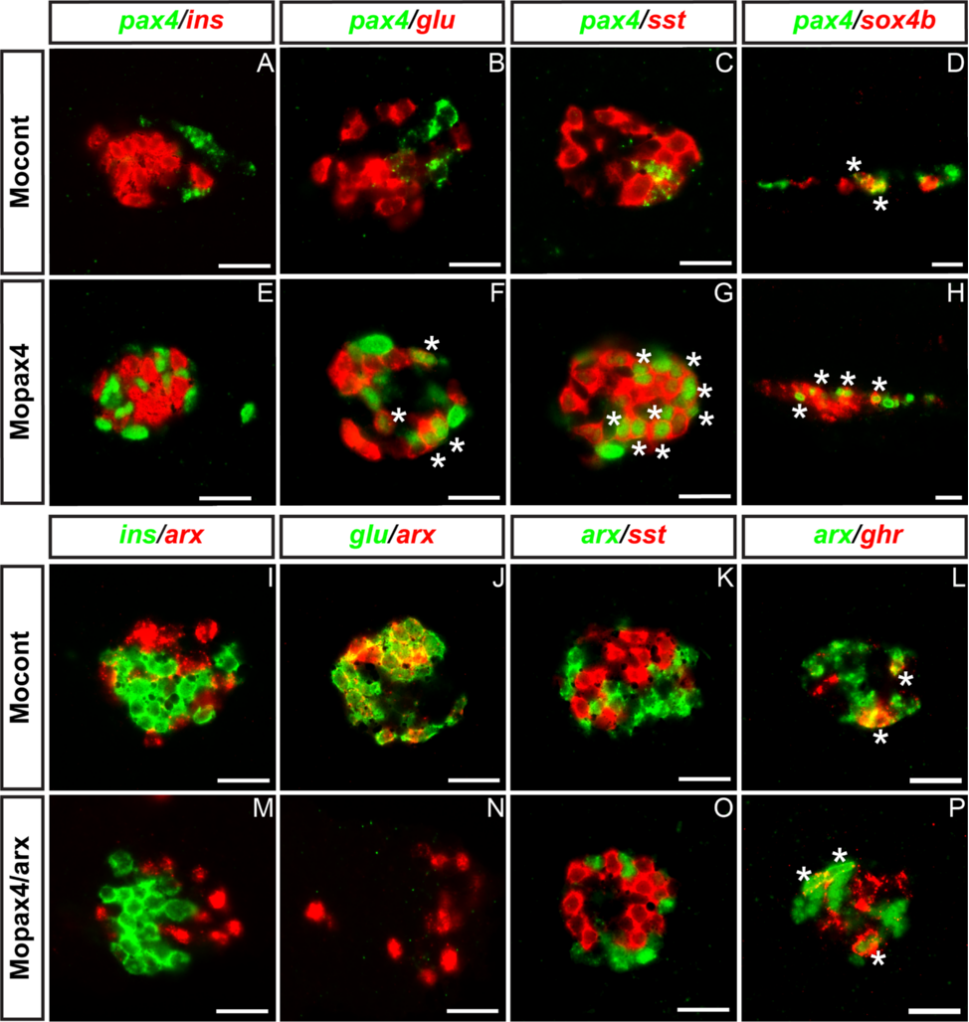
**Identity of *pax4+ *and *arx+ *cells following Pax4 and Arx inactivation. **(**A **– **F**) Double fluorescent WISH performed on 30 hpf embryos showing expression of *pax4* transcripts (green) in the different pancreatic cell types of control morphants (**A**, **C**, **E**, **G**) and in *pax4 *morphants (**B**, **D**, **F**, **H**). All panels display ventral vues except panels D and H which are lateral vues. (**I** – **N**) Confocal images of pancreatic area with *arx* probe and various pancreatic hormones at 30 hpf in control (**I**-**L**) and double *pax4/arx *morphants (**M**-**P**). Anterior part to the left. Scale bar = 20 μm. Asterisks (*) indicate double positive cells.

Our observation of the altered *arx* and *pax4* transcripts in the nucleus of pancreatic cells after their knock-down allows us to characterize these *arx*+ and *pax4*+ cells and thereby we determined whether their identity is modified after their respective knock-down. As already mentioned above, in wild-type embryos at 30 hpf, *pax4* is expressed in *sox4b*+ endocrine precursors, in some δ- and ε-cells and is excluded from α cells (see Figure 2 and Figure 8). In the *pax4* morphants, the expression profile of the altered *pax4* transcripts remained essentially the same as in wild-type except that they were also detected in the nuclei of α-cells (Figure 8A-H). These data suggest that, in the absence of functional Pax4 activity, the endocrine progenitors that should have expressed *pax4* can give rise to α-cells as well as to δ- and ε-cells. As *arx* gene transcription is strongly inhibited following *arx* knock-down, it was difficult to evaluate the identity of *arx*+ cells in *arx* morphants using the same strategy. To circumvent this problem, we analyzed instead the identity of *arx*+ cells in the double *arx;pax4* morphants where *arx* transcripts are easily detected. Like in the control embryos, *arx* transcripts were not detected in β- or δ-cells in the double morphants (Figure 8I,J,M and N). No glucagon expression could be detected confirming the efficiency of *arx* knock-down. Only a minority of *arx*+ cells expressed ghrelin, while the majority was not stained by any other of the hormone probes (Figure 8I-P). These data indicate that the fate of *arx*+ cells has not been switched to another cell type after *arx;pax4* knock-down and that these *arx*+ cells are probably blocked in their differentiation process toward α cells.

## Discussion

**Figure 9:**
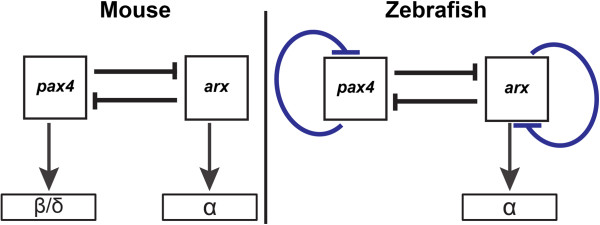
**Comparison of the *pax4 *and *arx *regulatory circuits in zebrafish and mouse. **A mutual repression between arx and pax4 is present in both species and α-cell differentiation relies on Arx activity. While PAX4 is required for β- and δ-cell formation in mouse, it is dispensable in zebrafish for that function. In zebrafish, Arx and Pax4 can repress their own expression. All interactions can be either direct or indirect.

Previous studies in mouse have shown that the balance of α-cells versus β/δ-cells is controlled by the antagonistic action of ARX and PAX4 homeodomain factors, ARX being required for α-cell identity and, inversely, PAX4 favoring β- and δ-cell fate at the expense of α-cells (see Figure 9) [27]. PAX4 is also sufficient in mice to convert α-cells to β-cells [19]. However, the lack of *PAX4* gene in birds and in some amphibians, which possess β-cells, raises the question of its original function in lower vertebrates such as fish. In this study, we show that *pax4* is not required for β- and δ-cell differentiation in zebrafish embryos but modulates the number of α-cells through repression of the *arx* gene (Figure 9). Zebrafish Arx is required for α-cell differentiation and has a repressive action on *pax4* gene expression, like in mouse. Thus, we can reasonably conclude that the mutual repression between Pax4 and Arx was established early in vertebrate evolution and that *pax4* was not required for β-cell differentiation in the first vertebrate species such as fish; the crucial role of Pax4 in β-cell differentiation could have appeared much later, possibly during mammals’ evolution.

Comparison of the peptidic sequence of zebrafish Pax4 with its human and murine orthologs reveals strong divergence beside the homeodomain and the paired domain. In contrast, the sequence of the closely related Pax6 proteins has been extremely conserved. Nevertheless, several observations indicate that the zebrafish Pax4 protein is the actual ortholog of the mammalian Pax4; indeed, i) 13 amino acid residues of the homeodomain and paired domain are common between the Pax4 proteins of different species while divergent in Pax6 proteins, ii) synteny is clearly identified between the vertebrate Pax4 genes, iii) the zebrafish *pax4* gene, like mouse *Pax4*, is specifically expressed in pancreatic endocrine precursors and not in other tissues, and iv) we observe an antagonism between the zebrafish *pax4* and *arx* genes as reported in mouse. Surprisingly, we could not detect any expression of zebrafish *pax4* in differentiating β-cells in the embryos as well as in the 5 dpf larvae (data not shown). As a second *pax4* paralog could not be identified in the zebrafish genome and as the efficiency of our *pax4* knock-down experiment was high, we can conclude that *pax4* is clearly not essential for the generation of β-cells in zebrafish embryos. In contrast to *pax4*, the pancreatic expression and function of *arx* gene seems to have been well maintained throughout vertebrate evolution. Indeed, this gene is expressed in all α-cells and is required for their differentiation as in mouse. As the analyses of *arx* and *pax4* morphants were performed in this study at 30 hpf, the function of *arx* and *pax4* genes was tested only for the pancreatic endocrine cells deriving from the dorsal pancreatic bud. Do the Arx and Pax4 factors have the same role in the second wave of endocrine cells appearing in the zebrafish larvae after 3 dpf and which derive from the ventral pancreatic bud? While our unpublished data show the absence of glucagon in a minority of *arx* morphants and the presence of secondary insulin cells in some *pax4* morphants at 5 dp, it is difficult to draw clear-cut conclusions as the knock-down efficiency decreases in the zebrafish larvae 4 days after morpholinos injection. Late function of *arx* and *pax4* genes will require the generation and analysis of *arx* and *pax4* mutants.

An interesting point raised by the present study is the observation that altering the splicing of a particular RNA causes its retention in the nucleus. Retention of incorrectly spliced transcript has been previously described in yeast and seems to be part of a quality control mechanism set up in eukaryotes to prevent the synthesis of truncated and potentially deleterious proteins [28,29]. The nuclear location of altered *pax4* and *arx* transcripts allowed us to characterize the *pax4*- and *arx*-deficient cells in morphants. Using this strategy, we could highlight that the cells expressing the altered *pax4* transcripts correspond to endocrine precursors, ε- and δ-cells like in control embryos, but also to α-cells in *pax4* morphants. *pax4* transcripts could not be detected in β-cells either in control embryos or in *pax4* morphants. This is in sharp contrast with data obtained in mouse and reinforces our conclusion that, in zebrafish, *pax4* is not involved in β-cell differentiation. *arx* was expressed in all α-cells and in some ε-cells in control embryos. Analysis of the double *arx/pax4* morphants revealed no change in the fate of the *arx*+ cells and rather indicated a blockage in their differentiation process towards α-cells. Such data are also contrasting with those reported in mouse where the pancreatic cells switch from α-cell toward β- or δ-cell fate in the *Arx* KO mice [12]. The reason for such a difference is unclear but a likely explanation could be again the lack of Pax4 function in β/δ fate decision in zebrafish. Indeed, if Pax4 was sufficient to specify β-cell fate in zebrafish like in mouse, the increased number of pax4-expressing cells in *arx* morphants should have resulted in additional β-cells. The nuclear retention of unspliced transcript is an useful phenomenon allowing to i) to easily verify the efficiency of the knock-down induced by morpholinos in a single zebrafish morphant, and ii) to identify and characterize the cells that should normally have expressed the studied factor. The blockage of mRNA nuclear export seems to occur only if an entire intron remains within the mRNA. Indeed, in other cases, morpholinos targeting exon-intron junctions can also lead to the use of neighbouring cryptic splicing sites or to the removal of whole exon(s), as previously shown for *pax6b* or *bmp2a*[13,30]. In these latter cases, we did not observe a nuclear retention of the altered transcripts (data not shown).

Our study also demonstrates a negative auto-regulation of Arx and Pax4 on their own gene expression. Indeed, the knock-down of *pax4* leads to an increase in the number of pancreatic cells expressing high level of altered *pax4* transcripts. Similarly, *arx* knock-down causes a striking increase in the level of *arx* transcripts in all tissues expressing this gene, except in the pancreas. Direct negative auto-regulation is a common feature found in many organisms allowing repressors to rapidly reach a steady-state in their expression level and preventing an excess of their expression. However in the pancreas, the levels of *arx* transcripts were dramatically reduced following *arx* knock-down, indicating that the negative feed-back was thwarted or blocked by another regulatory circuit. We demonstrated that this is due to the repressive action of Pax4 whose expression level is up-regulated in *arx* morphants. The feed-back loop of Arx on its own gene was further verified on a transgene driving GFP expression under the control of ARX regulatory sequence. These data demonstrate that the regulation occurs at the gene transcription level; if this effect is direct or mediated by (a) factor(s) regulated by Arx protein, is still unknown.

It is noteworthy to mention that, while both *pax4* and *arx* are negative regulators of each other and of their own gene, the transcription of these 2 genes is regulated in opposite ways after their respective knock-down: indeed, loss of Pax4 activity leads to an up-regulation of *pax4* transcripts while loss of Arx activity causes a down-regulation of *arx* transcripts in pancreatic cells. How to explain such opposite behaviors as the actions of both factors seems identical on each gene (see Figure 9B)? One explanation could be the different timing of their onset. Indeed, as *pax4* expression starts at 16 hpf long before *arx* which appears around 22 hpf, the knock-down of *pax4* leads to an increase of *pax4* transcription that cannot be thwarted by Arx between 16 and 22 hpf. In contrast, the knock-down of Arx activity will directly enhance *pax4* expression which will then represses *arx* gene expression. Interestingly, a negative autoregulation of human PAX4 gene has been previously reported through in vitro experiments [31]. Thus, it is likely that the auto-regulation we describe here for zebrafish *arx* and *pax4* genes exists in many vertebrates such as humans.

## Conclusions

Our study demonstrates that, on one hand, the role of Arx in α-cell differentiation has been maintained from fish to mammals, but on the other hand, Pax4 has no apparent function in the formation of β-cell in zebrafish embryos. Thus, we propose that PAX4 acquired its essential role in β-cells differentiation quite late in vertebrates’ evolution. This can explain why chicken or Xenopus generate β-cells although they do not possess any *PAX4* gene. Transcription factors shown to be crucial for pancreatic cell differentiation in mammals generally display conserved pancreatic expression in lower vertebrates such as zebrafish, suggesting that the regulatory network controlling pancreatic cell differentiation is similar across vertebrate species. The present study shows that there are nevertheless some differences in the action of particular transcription factors, like PAX4. Another zebrafish transcription factor could play the same role as the mammalian PAX4. One possible candidate is the Mnx1/Hb9 homeodomain protein which is required for β-cell differentiation in zebrafish [32] and has been recently shown to repress α-cell fate [33], as described for PAX4 in mouse. Further experiments will be required to test this hypothesis.

## Methods

### Zebrafish strain

Zebrafish (*Danio rerio*) embryos and adults (AB strain) were raised and cared for according to standard protocols [34]. Wild-type embryos were used and staged according to Kimmel et al. [35]. Animal care and experimental use were approved by the Animal Ethical Committee of University of Liège (File #1172).

### Cloning of the zebrafish *pax4* gene

The zebrafish *pax4* gene was identified by performing a TBLAST search on the Ensembl Genome Browser site (http://www.Ensembl.org) using the mouse Pax4 protein sequence. *pax4* cDNA was cloned by nested RT-PCR on mRNA extracted from 12 hpf to 31 hpf embryos using two different pairs of primers; the first pair corresponding to 5’-GGAGTGTAAATCAGCTGGGTGGTGTG (upstream primer) and 5’-GCTCCCTCCTCATCCTCGCTCTACG (downstream primer) and the second pair corresponding to 5’- CGGACGTCCTCTGCCTGTCTACAAGC (upstream primer) and 5’- GGTCAGCAGATCTGGATAAAGCCCAC (downstream primer). Amplification was obtained with 30 cycles of 10s at 94°C, 30s at 64°C (outer primers) or 60°C (inner primers), and 60s at 68°C, followed by a final 7min extension at 68°C. The 541pb product was then cloned in pGEM®-T Easy vector (Promega), and sequenced.

The 3’ end of the *pax4* gene was completed by 3’ RLM-RACE according to the supplier protocol (FirstChoice® RLM-RACE Kit, Ambion) on mRNA extracted from 24 hpf embryos. Briefly, mRNA were reverse transcribed using as primers the provided poly-dT 3’ RACE adapter oligonucleotide. The 3’ *pax4* cDNA sequences were amplified by performing a nested PCR using as 3’ primers the outer and inner 3’ adapters provided by the supplier and as 5’ primers the pax4 outer and inner primers (*pax4* outer primer: 5’-GGCGACTGAGGGAATGAGACC; pax4 inner primer: 5’- CCTGTGGGCTTTATCCAGATCT). The amplified cDNA was cloned in pCR®II-TOPO® vector (Invitrogen), and sequenced. The deduced pax4 mRNA sequence was deposited to NCBI Genbank (FJ713024.1).

### Single and double fluorescent whole-mount *in situ* hybridization

Single whole-mount *in situ* hybridizations were performed as previously described by Hauptmann and Gerster [36]. Anti-sense RNA probes were synthesized by transcription of cDNA clones with T7, T3 or SP6 RNA polymerase and using digoxigenin labelling mix (Roche) or DNP-11-UTP (TSA™ Plus system, Perkin Elmer). They were subsequently purified on NucAway spin columns (Ambion) and ethanol precipitated. Double fluorescent *in situ* hybridizations were carried out as described by Mavropoulos et al. [25]. The *pax4* probe was generated by linearization of the 3’-RACE-pCRII-TOPO plasmid with *Xba*I and transcription with SP6 RNA polymerase. The following probes were also used: *arx*[23], *nkx6.1*[37], *neuroD*[38], *glucagon*[39], *insulin*[40], *somatostatin*[39], *ghrelin*[41], *sox4b*[25], and *isl1*[42]. The cocktail of four hormones probes namely *glucagon*, *insulin*, *somatostatin* and *ghrelin* were used to analyse global hormone expression.

Cell counting for visible whole mount *in situ* hybridization was performed directly under the microscope by focusing successively on each layer of stained cells.

### Imaging

All visible whole mount *in situ* hybridization images were taken using a digital camera connected to BX60 Olympus Microscope. The Analysis® program (Soft Imaging System GmbH, Belgium) was then used for image processing. Confocal imaging was performed with a Leica TCS SP2 inverted confocal laser microscope (Leica Microsystems, Germany). Pictures were processed using Adobe Photoshop software and adobe illustrator for figure mounting.

### Morpholino design and injections

Anti-sense morpholino oligonucleotides were designed and purchased from Gene Tools (Philomath, OR). Knock-down of *arx* was achieved either by the morpholino Moarx targeting the intron 2 – exon 2 junction (5’-GCGTCATATTTACCTGGTGAACACA) or by the morpholino MoTarx blocking the translation (5’-TCGTCGTCGTACTGACTGCTC**ATG**T). Two splicing blocking morpholinos were used for pax4 knock-down: Mo1pax4 targeting exon 2 – intron 2 junction (5’-TAGCCTACACTTGGCACTTGATCTC) and Mo2pax4 targeting exon 1 – intron 1 junction (5’-AGGTGAGAAGTTTACCTTCAGTATT). The amount of morpholinos injected in each embryo was 2ng for Moarx, 1ng for MoTarx, 6ng for Mo1pax4. Double knock-down experiments were performed by injecting simultaneously 5ng of Mo1pax4 and 2ng of Moarx. All the morpholinos were diluted in Danieau solution containing Rhodamine dextran 0.5% from which 1nL was injected into the yolk of one-cell stage wild-type embryos. A standard control Morpholino (Mocont) that does not target any gene was also designed by Gene Tools and used as control (5’-CCTCTTACCTCAGTTACAATTTATA).

The confirmation of the *arx* negative autoregulation was performed by injecting the *arx* morpholinos and control morpholinos in a stable transgenic line Tg(ARX enhancer:GFP)(Ishibashi et al. manuscript in preparation). Injected and non injected eggs were raised in E3 solution at 28°C and GFP expression was analyzed in the embryos at 2 and 3 dpf using a Leica inverted fluorescent microscope. Settings for the microscope and camera software (Nikon) were kept constant for all images. Embryos were also imaged at 3 dpf using a Zeiss LSM710 confocal and Zen software with constant settings (Laser intensity, pinhole, detector gain, scan speed, temperature and objective). Fluorescence was recorded as a square 16-bit image with edge length of 1024 pixels, and an average of 90 slices per embryos were generated. A minimum of ten embryos per condition were scanned. Each stack was projected into a 2D image using a maximum intensity projection in image J software. Integrated fluorescent density was then evaluated for each projection in image J and analyzed with excel. A *t*-test was used to compare obtained data.

## Competing interest

The authors declare that they have no competing interests.

## Authors’ contribution

JD and VV performed the experiments and participated in the writing of the paper. EM, MI, JG and SR have performed data described in Additional file 5: Figure S5. IM, MV and BP supervised the work, analyzed the data and wrote the paper. All authors read and approved the final manuscript.

## Supplementary Material

Additional file 1: Figure S1 PAX4 and PAX6 peptidic sequence alignment. The residues identical in all species are in yellow boxes, and the conserved residues in the majority of sequences are shaded in blue. The paired domain and the homeodomain are indicated by a line. The asterisks indicate amino acid positions conserved in all PAX4 sequences and different in PAX6 sequences, and hyphens indicate gaps in the peptidic sequence. Hs: *Homo sapiens*, Mm: *Mus musculus*, Dr: *Danio rerio*. Click here for file

Additional file 2: Figure S2 Chromosomal locations of the zebrafish and human *PAX4/PAX6 *genes. Comparisons of the *PAX4 *locus as well as the *PAX6* locus in zebrafish and human showing synteny. *PAX4* genes are flanked by *SND1* and *LRRC4 *genes in all examined vertebrates. *FSCN3 *and *ARF5* genes are located downstream of *PAX4 *in human (and mammals) while *hgfa* and *cacna2d1 *genes are found downstream *pax4 *in zebrafish (and all examined fish species). Click here for file

Additional file 3: Figure S3 Disruption of *pax4 *and *arx *RNA splicing by morpholinos. Schematic representation of pax4 and arx pre-mRNA showing the exon-intron junctions recognized by Mo1pax4, Mo2pax4 and Moarx morpholinos. P’ and P” indicate the locations of primers used for RT-PCR analyses. Gels on left show the amplified cDNA from control and morphants revealing the presence of intronic sequences in pax4 RNA for morphants. No amplification could be obtained for arx RNA from arx morphants due to the insertion of the 1687 bp intron 2. Click here for file

Additional file 4: Figure S4 *arx *knock-down using the translation blocking morpholino MoTarx. Analysis by WISH of *glucagon *expression (A, B) and of *arx *expression (C,D) in control embryos (A,C) and in embryos injected with MoTarx morpholino (MoTarx) (B,D). Ventral view, anterior to the left of pancreatic area at 400X magnification p: pancreas. Note the loss of pancreatic *arx *expression in MoTarx morphants and the cytoplasmic localisation of arx transcripts in both control and morphants. Click here for file

Additional file 5: Figure S5 Expression of the Tg(ARX enhancer:GFP) transgene is increased by arx knock-down. Representative images of Tg(ARX_enhancer:GFP) transgenic embryos that have been injected with arx morpholino in comparison to uninjected embryos and embryos injected with a standard control morpholino. Fluorescent overview images were taken with an inverted microscope (A), while fluorescent intensities were measured by confocal microscopy (B) and quantified (C) as described in Materials and Method. A. Lateral view of 2 dpf Tg(ARX_enhancer:GFP) embryos injected with ARX morpholino (2ng), control morpholino (2ng) or uninjected. B. Maximum intensity projection of confocal stack of 3 dpf Tg(ARX_enhancer:GFP) embryos (dorsal view) injected with ARX morpholino (2ng), control morpholino (2ng) or uninjected. C. Fluorescent intensity measurements. Means of 10 embryos ± Standard Deviation. Different from control at *P < 0.001. Click here for file
