# Supplementary figures and images for: Pax4 is not essential for beta-cell differentiation in zebrafish embryos but modulates alpha-cell generation by repressing arx gene expression
Source: BMC Dev Biol. 2012 Dec 17;12:37. doi: 10.1186/1471-213X-12-37 (PMC3563606; doi:10.1186/1471-213X-12-37)

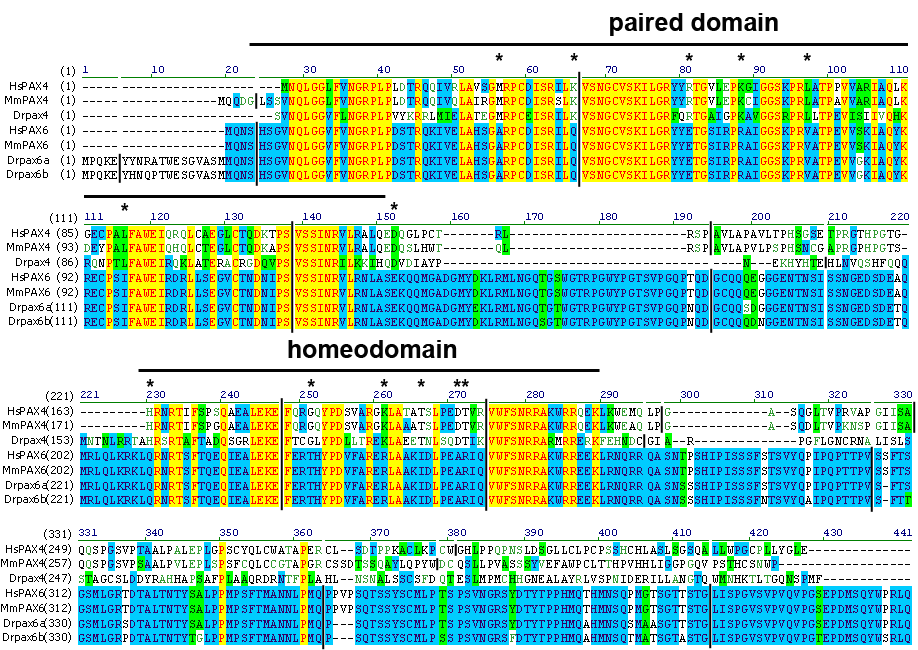

Supplement: Additional file 1: Figure S1 — PAX4 and PAX6 peptidic sequence alignment. The residues identical in all species are in yellow boxes, and the conserved residues in the majority of sequences are shaded in blue. The paired domain and the homeodomain are indicated by a line. The asterisks indicate amino acid positions conserved in all PAX4 sequences and different in PAX6 sequences, and hyphens indicate gaps in the peptidic sequence. Hs: Homo sapiens, Mm: Mus musculus, Dr: Danio rerio. [file 1471-213X-12-37-S1.docx]

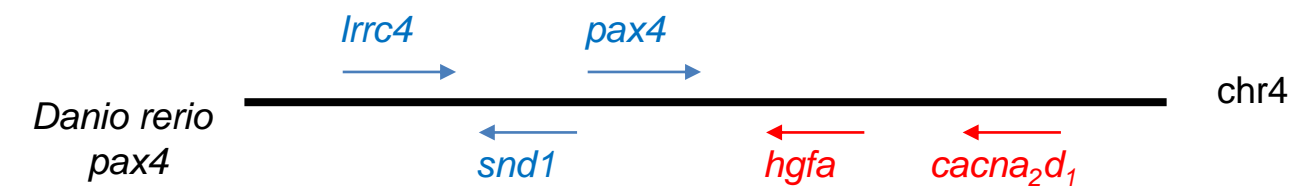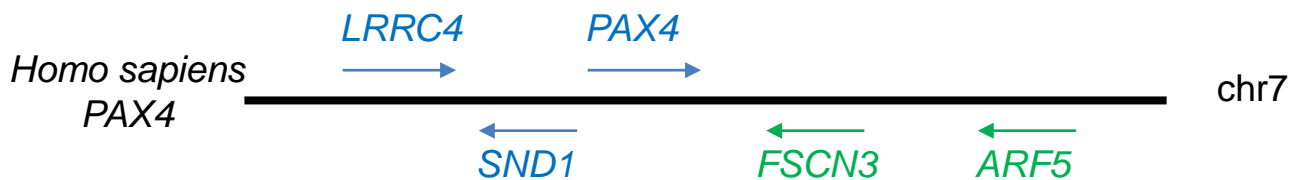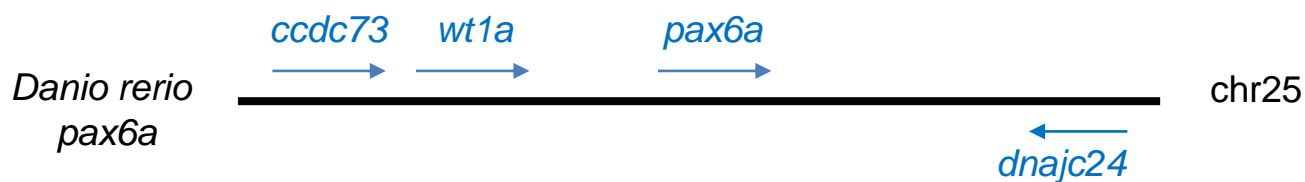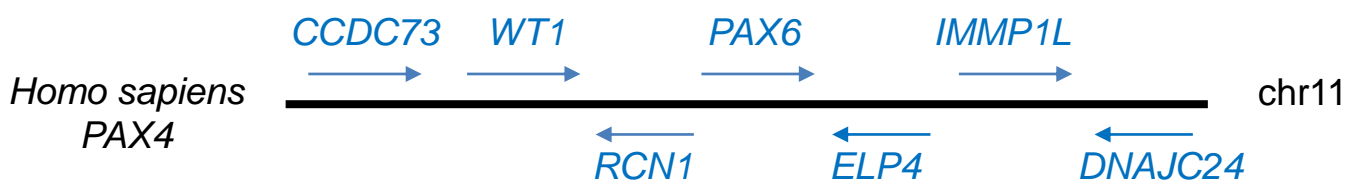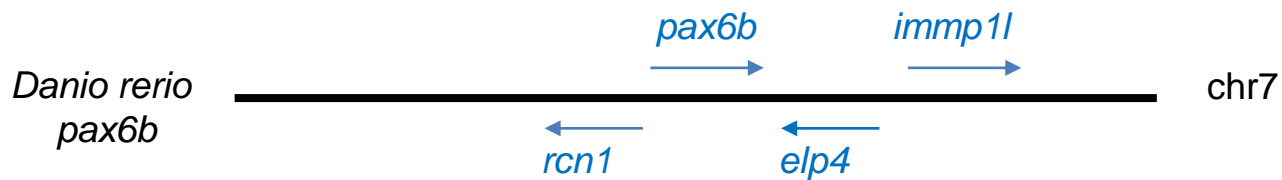

Supplement: Additional file 2: Figure S2 — Chromosomal locations of the zebrafish and human PAX4/PAX6 genes. Comparisons of the PAX4 locus as well as the PAX6 locus in zebrafish and human showing synteny. PAX4 genes are flanked by SND1 and LRRC4 genes in all examined vertebrates. FSCN3 and ARF5 genes are located downstream of PAX4 in human (and mammals) while hgfa and cacna2d1 genes are found downstream pax4 in zebrafish (and all examined fish species). [file 1471-213X-12-37-S2.pdf]

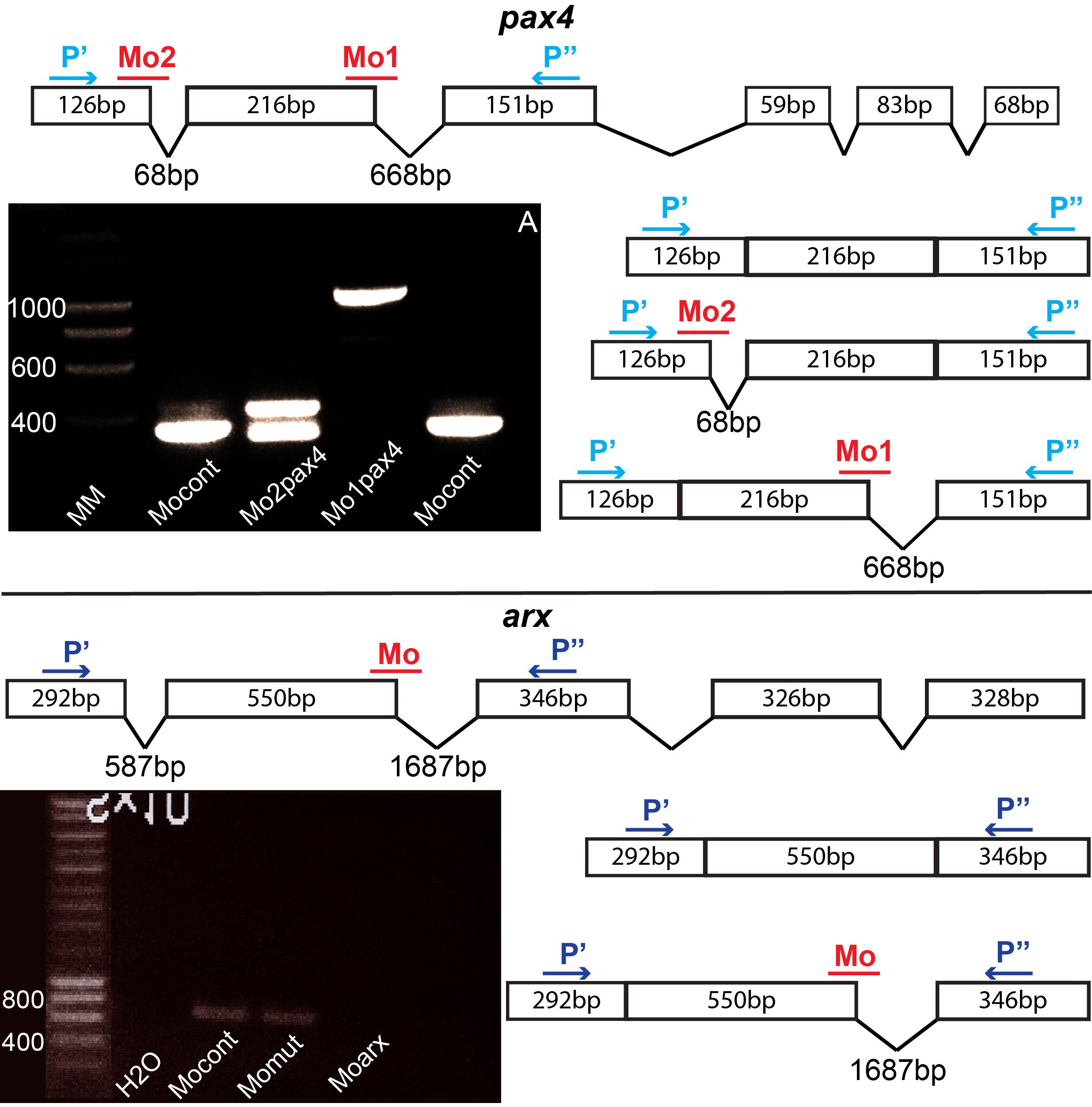

Supplement: Additional file 3: Figure S3 — Disruption of pax4 and arx RNA splicing by morpholinos. Schematic representation of pax4 and arx pre-mRNA showing the exon-intron junctions recognized by Mo1pax4, Mo2pax4 and Moarx morpholinos. P’ and P” indicate the locations of primers used for RT-PCR analyses. Gels on left show the amplified cDNA from control and morphants revealing the presence of intronic sequences in pax4 RNA for morphants. No amplification could be obtained for arx RNA from arx morphants due to the insertion of the 1687 bp intron 2. [file 1471-213X-12-37-S3.tiff]

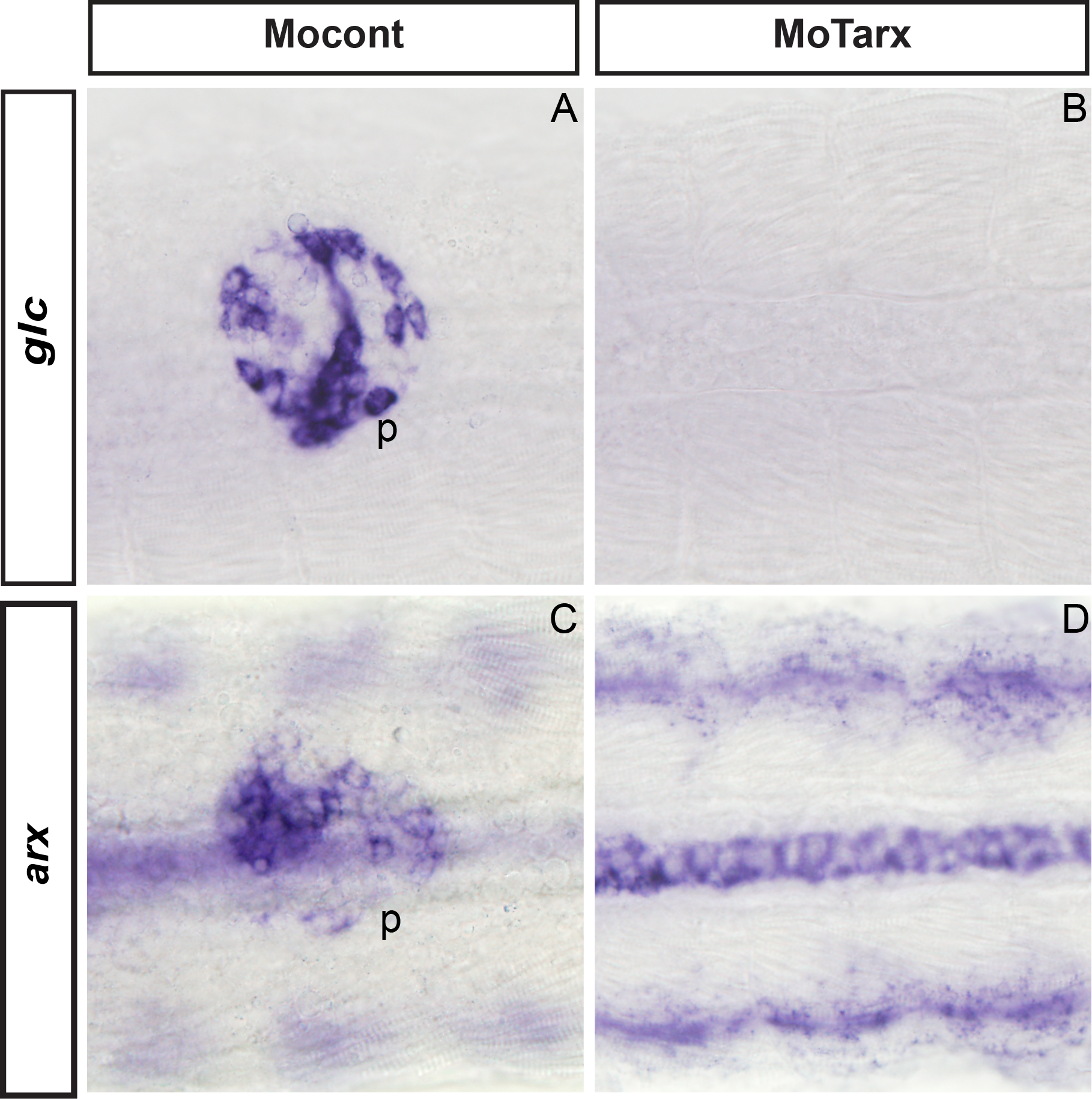

Supplement: Additional file 4: Figure S4 — arx knock-down using the translation blocking morpholino MoTarx. Analysis by WISH of glucagon expression (A, B) and of arx expression (C,D) in control embryos (A,C) and in embryos injected with MoTarx morpholino (MoTarx) (B,D). Ventral view, anterior to the left of pancreatic area at 400X magnification p: pancreas. Note the loss of pancreatic arx expression in MoTarx morphants and the cytoplasmic localisation of arx transcripts in both control and morphants. [file 1471-213X-12-37-S4.tiff]

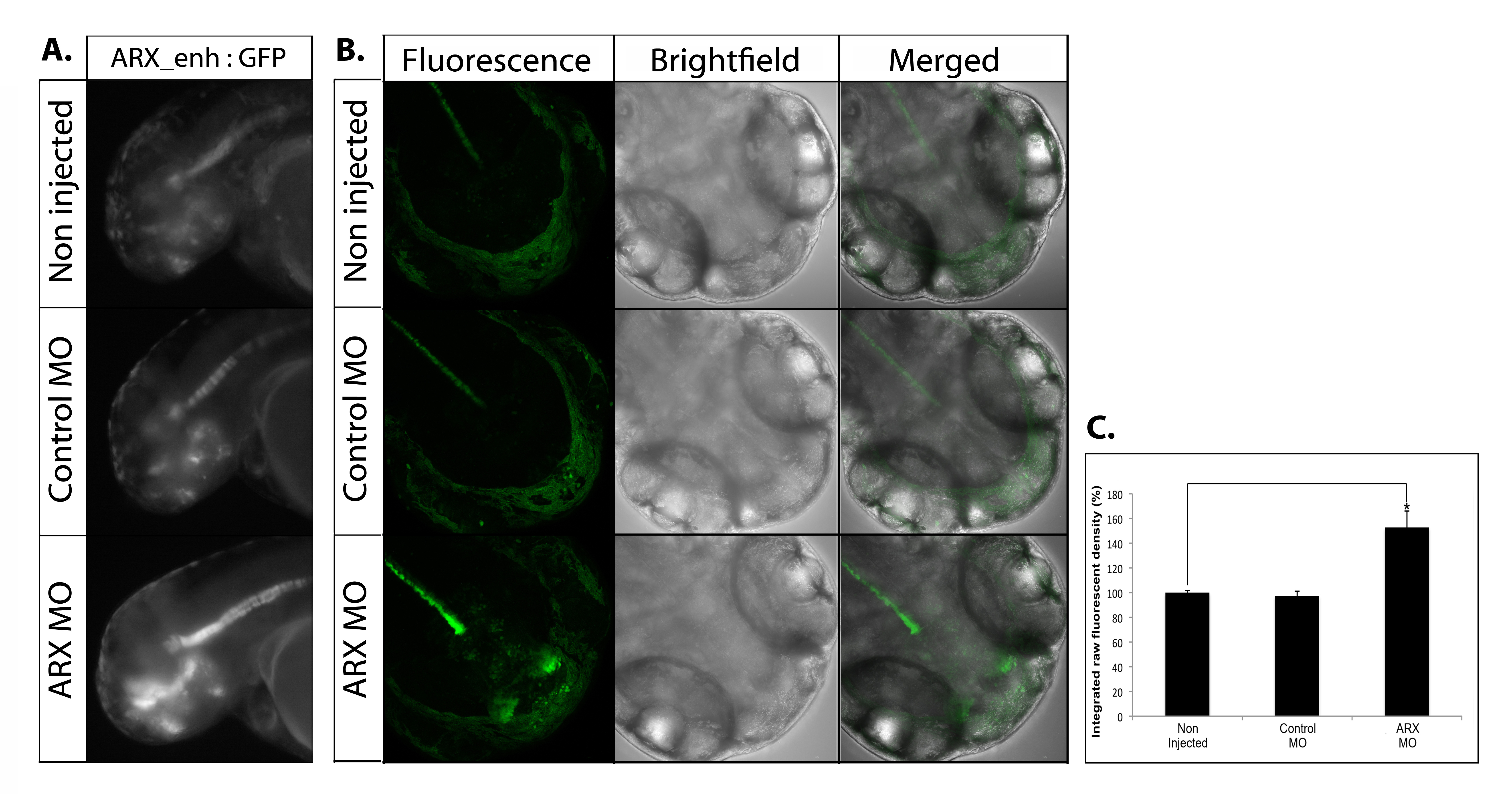

Supplement: Additional file 5: Figure S5 — Expression of the Tg(ARX enhancer:GFP) transgene is increased by arx knock-down. Representative images of Tg(ARX_enhancer:GFP) transgenic embryos that have been injected with arx morpholino in comparison to uninjected embryos and embryos injected with a standard control morpholino. Fluorescent overview images were taken with an inverted microscope (A), while fluorescent intensities were measured by confocal microscopy (B) and quantified (C) as described in Materials and Method. A. Lateral view of 2 dpf Tg(ARX_enhancer:GFP) embryos injected with ARX morpholino (2ng), control morpholino (2ng) or uninjected. B. Maximum intensity projection of confocal stack of 3 dpf Tg(ARX_enhancer:GFP) embryos (dorsal view) injected with ARX morpholino (2ng), control morpholino (2ng) or uninjected. C. Fluorescent intensity measurements. Means of 10 embryos ± Standard Deviation. Different from control at *P < 0.001. [file 1471-213X-12-37-S5.jpeg]
